# Spatial and temporal variation in bacterial–archaeal community and niche differentiation of denitrifying anaerobic methane-oxidizing microbes in grass carp (*Ctenopharyngodon idellus*) aquaculture ponds of Northern China

**DOI:** 10.3389/fmicb.2026.1782664

**Published:** 2026-04-10

**Authors:** Yan Chen, Zhe Pan, Zhaoxing Wu, Zhaohui Tian

**Affiliations:** 1 Fisheries Science Institute, Beijing Academy of Agriculture and Forestry Sciences, Beijing, China; 2 Ocean College, Department of Hebei Agriculture University, Qinhuangdao, Hebei, China

**Keywords:** aquaculture pond, archaea, bacteria, denitrifying anaerobic methane oxidation, sediment

## Abstract

**Objective:**

This study aimed to study the spatial and temporal variation in archaeal-bacterial community and niche differentiation of nitrite/nitrate-dependent anaerobic methane oxidation (N-/Nr-DAMO) microbes in the sediment of aquaculture ponds.

**Methods:**

Field sampling was carried out in May, July and September which represented different farming seasons in Northern China. The sediment samples were collected in the depth range of 0-15 cm at 5 cm intervals. The spatiotemporal distributions of sediment bacteria, archaea and N-/Nr-DAMO microbes, and characterized the niche differentiation of microbes were investigated in grass carp (*Ctenopharyngodon idella*) aquaculture ponds compared with a natural successional pond without human-induced practices. The copy numbers of total bacteria, total archaea, N-DAMO bacterial *pmoA* and Nr-DAMO archaeal *mcrA* were determined by qPCR, and the microbial community composition were determined by high-throughout sequencing analysis.

**Results:**

Bacterial and archaeal abundances and diversities were significantly higher in aquaculture ponds than in natural succession ponds, with distinct vertical distribution patterns. Specifically, the bacterial abundance and diverse, as well as the archaeal abundance showed decreasing trends with increasing sediment depth, whereas diversities of archaea displayed an opposite trend that increased with sediment depth in May and July. Bacterial co-occurrence networks were less complex in aquaculture ponds. In the case of N-DAMO bacteria, the copy numbers of the functional gene *pmoA*, the relative abundance, and diversity were higher, while their occurrences in bacterial networks were lower. The archaeal co-occurrence networks were more complexity and stability in aquaculture pond sediment. In the case of Nr-DAMO archaea, their relative abundances decreased significantly, and fewer Nr-DAMO archaea were present in the archaeal co-occurrence networks.

**Conclusion:**

The vertical profiles, as well as the aquaculture practices impact the bacterial and archaeal community structure which were shaped mostly by ORP and nutrient availability. In aquaculture ponds, bacterial co-occurrence networks were less complex, and fewer N-DAMO bacteria were presented in the bacterial co-occurrence networks. The archaea (e.g. desulphurizor, denitrifiers, methanogens) showed negatively correlated with respect to the distribution of Nr-DAMO archaea which were fewer presented in the archaeal co-occurrence networks. These results appeared that the niches of N-/Nr-DAMO microbes were suppressed in aquaculture ponds.

## 1 Introduction

In aquaculture pond (AP) ecosystems, production increases depend mainly on intensive management, including improvement in stocking density and feed input, and the amounts of uneaten feed and feces that settle in the sediments (Hou et al., 2022; Ling and Weimin, 2010). The accumulation of organic wastes in sediments carries the risk of endogenous water pollution, which poses a significant threat to the AP ecosystem and the surrounding environment. Bacteria and archaea are abundant and distributed throughout the sediment and play crucial roles in nutrient biogeochemical cycles and maintaining ecosystem stability (Beman et al., 2011; Chen and Wen, 2021; Xie et al., 2024). Archaea have been demonstrated to be indispensable for driving nutrient cycling through functional complementarity with bacteria.

Microbial communities can be detected conveniently and accurately using high-throughput sequencing techniques. In APs, large population sizes and rapid turnover rates lead to a predominant response of sediment microbes to these favorable habitats, a trait that contributes to their high diversity (Resende et al., 2015), and to variations in microbial composition of fermenters, denitrifiers, sulfate and iron (FeIII) reducers, methanogens, and methanotrophs (Bakar et al., 2015; Liu et al., 2024). For example, previous studies have shown that microbial diversities in APs are affected by some indicators related to trophic conditions. Feed input alters microbial compositions from aerobic and oligotrophic to anaerobic, fast-growing, and copiotrophic, leading to the loss of microbial community richness and diversity (Lin and Lin, 2022). Lin et al. (2025) reported that functional microbes in methane (CH_4_) generation/oxidation and nitrification/denitrification are more sensitive to changes in microbial network complexity and stability in APs. The high turnover rates of microbial communities are more sensitive than single-organism species, which may provide a more comprehensive indicator for risk assessment and serve as a complement to potential recommendations for AP restoration.

CH_4_ is the second most potent greenhouse gas; its atmospheric concentration has risen to 1946 ppb, accounting for approximately 20% of anthropogenic global warming (NOAA, 2026). Aquaculture pond sediments are a reported “hot spot” for CH_4_ emissions (Lv et al., 2025; Liu et al., 2024). Zhang et al. (2026) reported that aquaculture systems account for <2% of the global lentic habitat area, contributing to about 8.8% of total CH_4_ emissions. CH_4_ emissions in aquatic ecosystems are controlled by microbes. Biogenic CH_4_ is produced by methanogenic archaea under anaerobic conditions, and oxidized by methanotrophs, which use CH_4_ as a sole energy source and consume over 90% of CH_4_ before it reaches the atmosphere (Franchini et al., 2015; Wang et al., 2022). Traditionally, aerobic methanotrophic communities utilize oxygen (O_2_) as the electron acceptor, oxidizing CH_4_ to carbon dioxide (CO_2_), and have been considered the dominant way for CH_4_ reduction (Reis et al., 2020; Yang et al., 2023). However, the continuous flood conditions in freshwater ecosystems also create anoxic conditions, allowing for the anaerobic oxidation of CH_4_ (AOM), which is coupled with sulfate (SO_4_^2−^) (Timmers et al., 2016), nitrate (NO_3_^−^) (Haroon et al., 2013), nitrite (NO_2_^−^) (He et al., 2015), Fe^3+^ (Rooze et al., 2016), Mn^4+^ (Ettwig et al., 2016), and humic substances (Liang et al., 2024), as the electron acceptors to oxidize CH_4_. Anaerobic oxidation has been confirmed to be a significant CH_4_ sink in freshwater ecosystems, efficiently oxidizing 200 Tg/yr. of endogenous CH_4_ emissions globally (Fan et al., 2020).

The denitrifying anaerobic methane oxidation (DAMO) identification are mediated by the *Methylomirabilota* (previously NC10 phylum) bacteria, which are represented by “*Candidatus Methylomirabilis oxyfera*-like” bacteria (*M. oxyfera*) that utilize NO_2_^−^ (N-DAMO) or archaea in the genus candidate *Methanoperedens* sp. (also known as ANME-2d, Anaerobic Methanotrophic Archaea Group 2d archaea), represented by “*Candidatus Methanoperedens nitroreducens*-like” archaea (*M. nitroreducens*) that use NO_3_^−^ as the electron acceptors (Nr-DAMO) to oxidize CH_4_ (Ettwig et al., 2010; Haroon et al., 2013; Jiang et al., 2023; Hu et al., 2025). Using stable isotope and molecular biology techniques in freshwater ecosystems, past studies have reported that N-/Nr-DAMO activity and the occurrence of bacteria closely related to *M. oxyfera*, as well as archaea closely related to *M. nitroreducens* (Ettwig et al., 2010). N-/Nr-DAMO processes were shown to play important roles in coupling CH_4_ emission mitigation with nitrogen removal in inland aquatic ecosystems (Bai et al., 2024; Fu et al., 2017; Shen et al., 2014). For example, the nitrite–DAMO process was estimated to be responsible for an approximately 2–6% reduction of the annual CH_4_ emissions (4.1–6.1 Tg) in freshwater wetlands (Hu et al., 2014) and about 11.2% reduction of the annual CH_4_ emissions (0.91 Tg) in Chinese paddy fields (Yang et al., 2024). The *M. nitroreducens* can provide nitrite (NO_2_^−^) to *M. oxyfera* bacteria, and the coexistence of these two N-/Nr-DAMO microbes has been widely documented (Fu et al., 2017; Hu et al., 2025; Shen et al., 2017; Wang et al., 2022). The distribution of N-/Nr-DAMO activities and the functional microbes can be affected by vertical sediment profiles (Yu et al., 2023), trophic levels (carbon and nitrogen levels) (Fu et al., 2017; Wang et al., 2022), and climatic factors (temperatures) (Wang et al., 2026), which were explained by environmental gradients and resource availability in the sediment profiles. In addition, the N-/Nr-DAMO microorganisms have been reported to undergo sustained depression in metal-contaminated (e.g., inorganic copper) freshwater systems (Xia et al., 2024). In the case of human-induced ecosystems, nitrogen deposition, CH_4_ emission, and anoxic zones in freshwater APs provide favorable conditions for N-/Nr-DAMO microbes as reported previously (Wang et al., 2026). Whereas nutrient enrichment, disinfectant use, and pathogen presence create more complex environmental conditions (Fan et al., 2019; Zhang et al., 2025), relevant scientific data remain scarce.

The feed input has been reported to trigger rapid changes in sediment physicochemical properties, microbial community, and greenhouse gas (GHG) production in APs (Lin and Lin, 2022; Liu et al., 2024; Lin et al., 2025). Thus, some microorganisms inevitably exhibit synergy/promotion and antagonism/inhibition with respect to N-/Nr-DAMO microbial growth, affecting their niche differentiation, especially during the late culture stages (maturation and harvesting periods). This study investigated the spatiotemporal distributions of sediment bacteria, archaea, and N-/Nr-DAMO microbes, and characterized the niche differentiation of microbes between three grass carp (*Ctenopharyngodon idella*) APs and a natural successional pond without human-induced practices in North China. This information may help in understanding the microbial contributions to sediment functions and N-/Nr-DAMO dynamics, offering insights into strategies for the concurrent reduction of nitrogen and CH_4_ emissions in APs.

## 2 Methods

### 2.1 Sediment sampling

The study aquaculture farm was located in Shunyi District, Beijing (39°15′ and 115°15′E) in 2023. This farm has carried out grass carp aquaculture for more than 20 years. The aquaculture practices lasting from April to October. Field sampling was carried out in May, July, and September, representing different farming seasons in Northern China. Water and sediment samples were collected from three grass carp APs (area, 0.67 ha; depth, 1.6 m) with a stocking density of 22,522 kg/ha and 3% of the fish weight feed input as well as natural succession pond (NP, 0.60 ha in area, 1.6 m in depth) stocked with omnivorous fish without human-induced practices. The temperature (T), pH, dissolved oxygen (DO), and conductivity in the surface (0–0.5 m), middle (0.5–1.0 m), and bottom (1.0–1.5 m) of the water column were measured *in situ* using a multi-parameter water quality probe (YSI 6600, Yellow Springs, Ohio, United States) during sampling. The collected water samples were stored in plastic bottles and then transferred to the laboratory for further analysis. The surface sediments (0–15 cm) exhibit high permeability, were subject to rapidly changing conditions, and were sensitive to hydrodynamics. These may result in significant separation of microbial community structure, even within smaller depth intervals (5 cm). Therefore, sediments were sampled at a depth of 0–15 cm at 5 cm intervals using a customized sediment push-corer (5 cm in diameter). The corresponding samples of the sediment layers were named AP_5, AP_10, and AP_15 in APs, and NP_5, NP_10, and NP_15 in the natural succession pond, respectively. The redox potentiometer (YHBJ-262, Leimci, China) was used *in situ* to determine the oxidation–reduction potential (ORP, which reflects the redox state of sediments) and temperature of each sediment sample.

### 2.2 Determination of physical and chemical factors

Water quality indexes, including total nitrogen (TN), ammonium nitrogen (NH_4_^+^-N), nitrite nitrogen (NO_2_^−^-N), and nitrate nitrogen (NO_3_^−^-N), were determined by spectrophotometry methods. Dissolved organic carbon (DOC) content was determined using a total organic carbon (TOC) analyzer (Vario TOC cube Total Organic Carbon TOC analyzer, Elementar, Germany) after filtering the water through a Whatman filter membrane (0.22 μm). Chlorophyll *a* content was determined by spectrophotometry described in previous study (Liu et al., 2024).

The sediment samples were air-dried, and the water content was calculated. The sediment samples were ground and sieved through a 100-mesh. The samples were extracted with 2 mol/L potassium chloride (sediment/water = 1:5) and the concentration of TN, NH_4_^+^, NO_2_^−^, and NO_3_^−^ was determined by spectrophotometry. The TOC content in the sediments was determined using a TOC analyser (Vario TOC cube Total Organic Carbon TOC analyser, Elementar).

### 2.3 DNA extraction, amplification, and high-throughout sequencing analysis

Genomic DNA was extracted from 0 to 5 cm (AP_5), 5–10 cm (AP_10), and 10–15 cm (AP_15) sediment layers of APs and 0–5 cm (NP_5), 5–10 cm (NP_10), and 10–15 cm (NP_15) sediment layers of NP during different farming seasons. Six parallel sediment samples were used for the microbial DNA extraction. The microbial DNA extraction from 0.5 g of well-mixed sediment samples was performed according to the manufacturer’s instructions using the PowerSoil DNA isolation kit (MO BIO, United States). The DNA purity and concentration were checked by 1.2% agarose gel electrophoresis and spectrophotometry (DS-11 UV–Vis spectrophotometer, DeNovix, United States).

The primer pairs 515F (GTGYCAGCMGCCGCGGTAA)/806R (GGACTACNVGGGTWTCTAAT), and Arch519F (CAGCCGCCGCGGTAA)/Arch915R (GTGCTCCCCCGCCAATTCCT) were used to amplify the V3–V4 regions of bacterial and V4–V5 regions of the archaeal 16 s rRNA genes, respectively (Yu et al., 2023; Liu et al., 2024). The concentrations of purified PCR amplicons were determined using Quant-iT PicoGreen dsDNA Assay Kit. Sequencing libraries were constructed using the TruSeq Nano DNA LT Library Prep Kit for Illumina, according to the manufacturer’s instructions, and index codes were added. Sequencing was performed on the Illumina MiSeq PE300 platform (Personalbio Bio-Pharm Technology Co., Ltd., Shanghai, China). Raw sequence data obtained in this work have been submitted to the National Genomics Data Center (NGDC) Genome Sequence Archive (GSA) database under the accession numbers CRA031053 for archaea and CRA031059 for bacteria.

The sequencing data were quality filtered, denoised, and merged, and chimeras were removed with the Divisive Amplicon Denoising Algorithm 2 method (DADA2) using the Quantitative Insights into Microbial Ecology (QIIME2, version 2019.4) pipeline, through which any low-quality sequences were discarded (Callahan et al., 2016). Specifically, reads with >25 quality score and fragments larger than 100 bp were retained. Merge the obtained sequences with 100% sequence similarity to generate amplicon sequence variants (ASVs) and the abundance data table. The taxonomic assignment of each ASV was annotated using the QIIME feature-classifier classify-sklearn module and the SILVA database version 138 (Quast et al., 2013). High-quality *M. oxyfera-like* bacterial sequences and *M. nitroreducens-like* archaeal sequences were extracted from the *Methylomirabilaceae* and the genus of “*Candidatus Methanoperedens*,” respectively.

Alpha diversity analysis was performed using QIIME2. Sequencing depth was assessed with rarefaction curves. Subsequently, all samples were rarefied to 95% of the minimum library size to normalize sequencing depth. The alpha-diversity indices were calculated to compare species richness and evenness across samples. The beta-diversity analysis was conducted through nonmetric multidimensional scaling (NMDS) with the Bray Curtis dissimilarity estimates, which were drawn using the Vegan software package of R software version 2.15.3. Analysis of similarity (ANOSIM) was further conducted to analyze the microbial differences between ponds or among sediment depths. In addition, the linear discriminant analysis effect size (LEfSe) was used to identify the taxa differences from phylum to genus between NP and APs or among depth layers. Based on the extracted sequences of *M. oxyfera*-like bacterial sequences and *M. nitroreducens*-like archaeal sequences in Silva database, the α-diversity of the Shannon index, the Simpson and Chao1 estimators were calculated.

### 2.4 Real-time quantitative PCR assay (qPCR)

The abundances of total bacterial, total archaeal, N-DAMO bacterial *pmoA*, and Nr-DAMO archaeal *mcrA* were determined using the real-time quantitative PCR (qPCR) assay with primer sets 515F/907R, Ar109F/Ar344R, cmo182/cmo568, and McrA159F/McrA345R, respectively. The qPCRs were performed in 20 μL reaction volumes in ABI PRISM® 7500 Sequence Detection System (Applied Biosystems, United States) using TB Green Premix Ex Taq (Tli RNaseH Plus; Takara, Dalian, China). The details of the primers and PCR reaction system are listed in Supplementary Table S1. Prior to sample determination, standard curves were constructed from a series of 10-fold concentration-gradient dilutions (10^2^–10^8^) of the known copy numbers of standard plasmids containing the target genes. The standard curve R^2^ value was greater than 0.98, and the amplification efficiency was between 90 and 110%. Triplicate qPCR analyses were performed for each sample, and the negative controls were included in all runs. The gene abundance was expressed in copies/g (dry weight).

### 2.5 Statistical analysis

The statistical analyses of gene abundances and physicochemical factors in the sediment were implemented in SPSS version 22.0 (IBM Corporation, NY, United States). Prior to statistical analyses, the Kolmogorov–Smirnov and Levene’s tests were used to assess the normality of distributions and homogeneity of variance, respectively. To satisfy the assumptions of the statistical analyses, the abundances of total bacteria, total archaea, N-DAMO bacterial *pmoA* gene, and Nr-DAMO archaeal *mcrA* genes were first log-transformed. Differences in sediment physicochemical characteristics, microbial abundances, and diversity indexes between APs and NP and among sediment depth were assessed using the two-way analysis of variance (ANOVA), followed by the least significant difference (LSD) test and the independent-samples *T*-test for multiple comparisons. Main-effect analysis examines the influence of a single independent variable (pond type or sediment depth) on a dependent variable (e.g., sediment physicochemical properties), without considering other independent variables. The Mantel tests and redundancy analysis (RDA) were used to determine the relationships between sediment environmental factors and microbial communities.

The bacterial and archaeal networks of APs and NP were performed using the Molecular Ecological Network Analyses (MENA) from the obtained ASVs, with the subcommunities of three different months analyzed separately. Only the ASVs occurring in more than 50% of all samples were retained in the networks to reduce false-positive results and minimize dataset complexity. The species co-occurrences included only strong with coefficient (|*r*| > 0.8) and statistically significant (*p* < 0.01, fdr-adjusted) Spearman’s correlations in the networks. The nodes in the networks represented individual ASVs, and the edges represented the connections of the co-occurrence networks. The numbers of nodes and edges, the average degree, and the average clustering coefficient (avgCC) were calculated and then visualized using the interactive Gephi 0.9.2 platform. Keystone species were identified using thresholds for intra-module (*Zi* ≥ 2.5) and inter-module (*Pi* ≥0.62) connectivity.

## 3 Results

### 3.1 Physicochemical properties of samples

**Figure 1:**
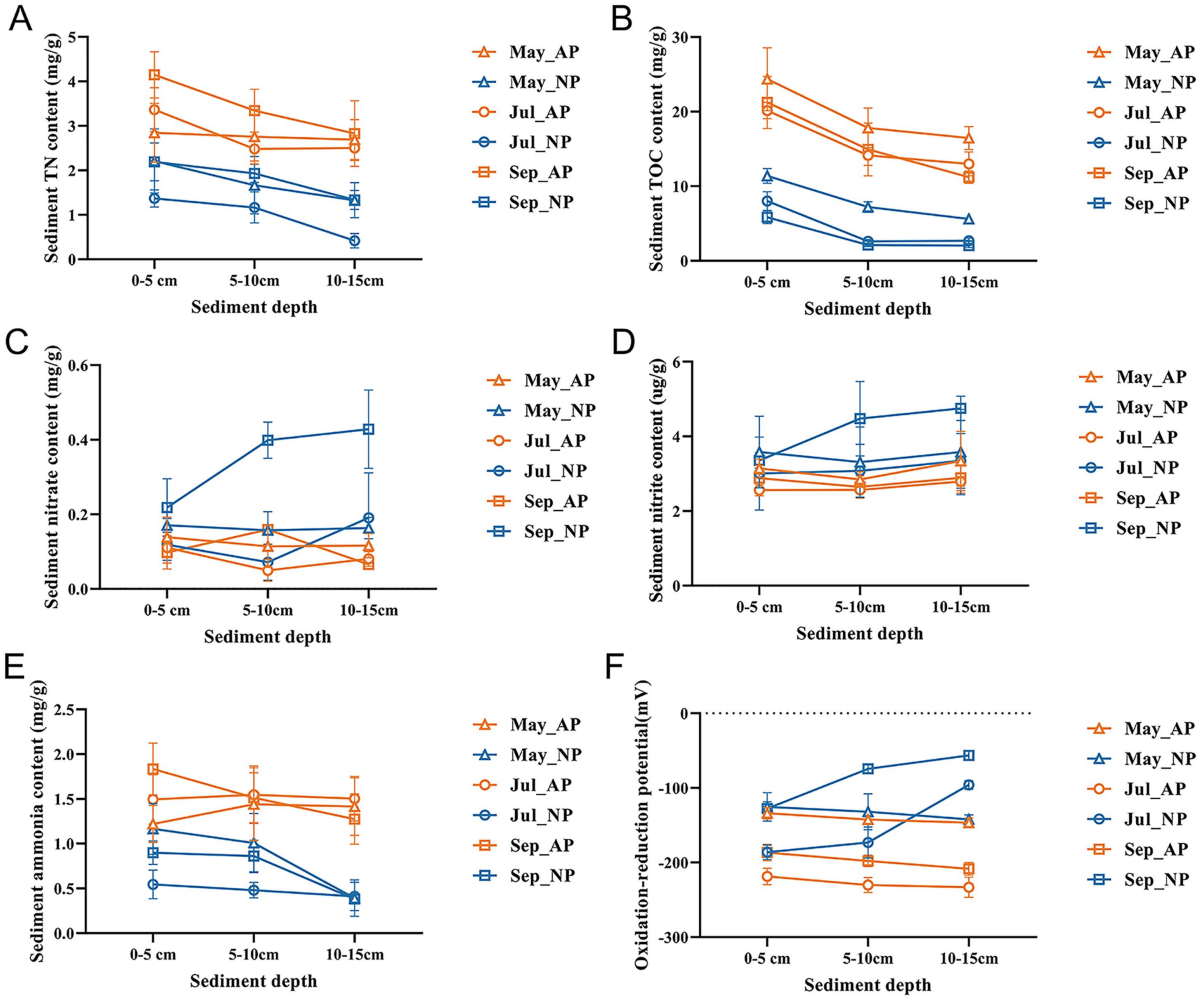
Vertical variation patterns of physicochemical characteristics **(A)** TN, total nitrogen; **(B)** TOC, total organic content; **(C)** nitrate; **(D)** nitrite; **(E)** ammonia; **(F)** ORP, oxidation-reduction potential in aquaculture sediment samples for different depths and farming seasons.

Variations in physicochemical properties in the sediments are shown in Figure 1 and Supplemental Data S1, and the statistical analysis through two-way ANOVA of APs and NP in different sediment depths are shown in Supplementary Table S2. In May, the NH_4_^+^ and TOC contents were significantly affected by the ponds and sediment depth. According to the multiple comparisons, TOC content in the surface 0–5 cm sediment demonstrated significantly higher levels in APs compared with NP. The TOC and TN contents (0–15 cm layers), and the NH_4_^+^ content (5–15 cm layers) were significantly higher in the sediment of APs compared with the NP (Figure 1). In July, sediment depth and pond type significantly affected TN, NO_3_^−^, NH_4_^+^, TOC, and ORP. The TOC, TN, NH_4_^+^, and NO_3_^−^ contents were higher, while the NO_2_^−^ and ORP were lower in APs compared with the NP. Regrading vertical distribution, the contents of TN and TOC (NP and APs), as well as NO_3_^−^ (APs), were significantly higher in the surface 0–5 cm sediment layer (*p* < 0.05). In September, the NO_3_^−^ and TOC contents were also influenced by the interaction between pond type and sediment depth (*p* < 0.05). Significantly higher levels of TOC, TN, and NH_4_^+^ contents, whereas significantly lower levels of NO_2_^−^, NO_3_^−^, and ORP were observed in APs. The spatial distributions of TN, TOC, and NH_4_^+^ were shaped by sediment depth, showing significantly higher concentrations in the 0–5 cm surface sediment layer in both ponds (*p* < 0.05).

### 3.2 Copy numbers of total bacteria, total archaea, N-DAMO bacterial *pmoA* and Nr-DAMO archaeal *mcrA*

**Figure 2:**
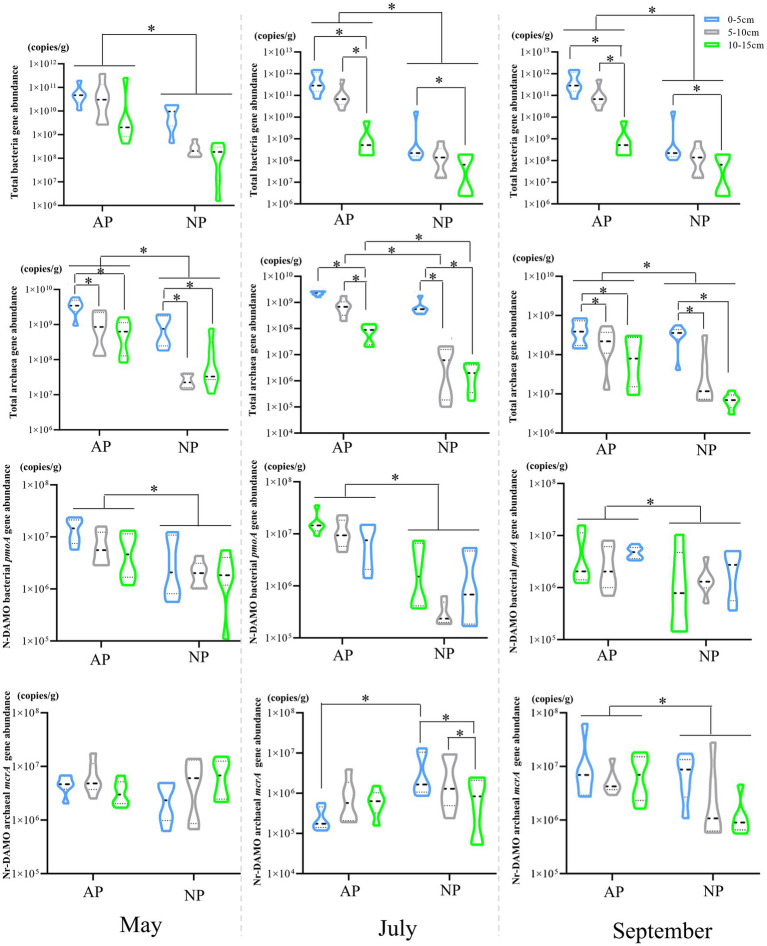
Copy numbers of the total bacteria, total archaea, N-DAMO bacterial pmoA and Nr-DAMO archaeal mcrA in aquaculture sediment samples for different depths and farming seasons. The *P* < 0.05 is considered statistically significant and is indicated by ‘*’.

The ranges of copy numbers for total bacteria, total archaea, N-DAMO bacterial *pmoA*, and Nr-DAMO archaeal *mcrA* are shown in Figure 2, and the interactions of pond type and sediment depth that affect the abundances are shown in Supplementary Table S3. In May and September, the abundances of these genes were not influenced by the interaction of pond type and sediment depth; however, they were significantly affected by both factors, according to the main-effect analysis (Supplementary Table S3). In July, the abundances of total bacteria, archaea, and Nr-DAMO archaeal *mcrA* were significantly influenced by the interaction between pond type and sediment depth (Supplementary Table S3). The abundances of total bacteria and archaea were significantly higher in APs. Regarding vertical distribution, the total bacterial and archaeal abundances showed similar patterns, with total bacterial abundances were significantly higher in the 0–5 cm sediment layer in July and September, whereas the total archaeal abundances decreased significantly with depth across all sampling periods (Figure 2). The N-DAMO bacterial *pmoA* gene abundances were increased significantly in APs (Figure 2), although no significant vertical differentiation was observed. The abundances of Nr-DAMO archaeal *mcrA* gene varied in different farming seasons. In May, there were no significant differences across sediment depth and pond type. In July, the abundances of the Nr-DAMO archaeal *mcrA* genes in the 0–5 cm sediment layer were significantly higher in APs. The vertical distribution of Nr-DAMO archaeal *mcrA* gene in the NP showed significantly higher abundances in the 0–10 cm sediment layer (Figure 2). Unlike the NP, there were no significant impacts of sediment depth on the Nr-DAMO archaeal *mcrA* gene abundances in the APs (Supplementary Table S3). In September, the abundances of the Nr-DAMO archaeal *mcrA* genes were notably higher in APs, with no significant differences were detected in the sediment depth profile.

### 3.3 Vertical distributions of microbial community alpha- and beta-diversity

The numbers of bacterial and archaeal ASVs and diversities are shown in Tables 1, 2, respectively. In May, the numbers of bacterial and archaeal ASVs increased along sediment depth in NP, whereas no clear vertical trend was observed in the APs sediment. The diversities of both bacteria and archaea were significantly higher in APs (Table 2). The microbial diversities showed similar patterns, with significant decrease in bacterial diversities, whereas significant increases in archaeal diversities occurred along sediment depth. In July, the bacterial and archaeal ASVs numbers increased with sediment depth in NP, while no apparent vertical differentiations were observed in the APs. The diversities of bacteria and archaea demonstrated no significant differences in vertical sediment profiles, while the archaeal diversities of APs were significantly higher at each sediment depth (Table 2). In September, the bacterial ASV numbers increased along sediment depth in both ponds, whereas archaeal ASVs numbers showed a reverse trend between NP and APs (Table 1). The bacterial and archaeal diversities demonstrated similar vertical patterns, decreasing with sediment depth. Compared with the NP, the bacterial diversities decreased significantly in 5–10 cm and increased in 10–15 cm sediment layers, and the archaeal diversities increased significantly at each depth in APs (Table 3).

**Table 1:** Number of total bacterial, *Methylomirabilis oxyfera*-like bacteria, total archaeal, and *M. nitroreducens*-like archaea sequences in each sample obtained by high-throughput 16S rRNA gene sequencing.

Sediment samples	No. of bacterial sequences	No. of *M. oxyfera*-like bacteria sequences	Percentage of *M. oxyfera*-like bacteria sequences (%)	No. of archaeal sequences	No. of *M. nitroreducens*-like archaea sequences	Percentage of *M. nitroreducens*-like archaea sequences (%)
M_NP_5	62,036	212	0.34	54,740	1,646	3.00
M_AP_5	74,869	372	0.49	43,747	301	0.65
M_NP_10	90,682	257	0.28	66,047	38,601	57.8
M_AP_10	73,161	306	0.42	44,318	5,352	11.4
M_NP_15	93,093	194	0.22	70,180	39,446	55.3
M_AP_15	78,285	301	0.39	47,560	7,395	14.1
J_NP_5	74,463	205	0.28	55,617	1,656	3.12
J_AP_5	80,126	489	0.59	47,241	100	0.20
J_NP_10	87,077	175	0.21	59,643	40,811	67.3
J_AP_10	85,217	403	0.48	43,517	657	1.41
J_NP_15	93,639	195	0.22	67,605	38,144	54.8
J_AP_15	82,358	307	0.37	47,864	6,768	12.8
S_NP_5	72,440	220	0.30	70,894	8,179	11.2
S_AP_5	79,352	499	0.61	57,354	836	1.50
S_NP_10	100,178	325	0.32	57,361	33,172	54.1
S_AP_10	89,749	247	0.28	41,177	3,481	8.21
S_NP_15	85,783	242	0.28	80,834	57,355	70.8
S_AP_15	82,794	357	0.43	50,462	1,930	3.62

**Table 2:** Variations in Chao1 and Shannon index of bacterial and archaeal communities in sediments at different depths in May, July, and September.

Sampling sites	Bacteria		Archaea	
	Chao1	Shannon	Chao1	Shannon
NP_M_5	3946.5 ± 292.5^Ba^	10.2 ± 0.27^Aa^	650.7 ± 83.3^Aa^	5.41 ± 0.39^Bb^
AP_M_5	4478.9 ± 426.0^Aa^	10.4 ± 0.14^Aa^	672.3 ± 71.4^Ab^	6.41 ± 0.39^Aa^
NP_M_10	3157.1 ± 411.8^Bb^	9.49 ± 0.51^Bb^	716.5 ± 152.1^Aa^	6.10 ± 0.41^Ba^
AP_M_10	3890.2 ± 370.2^Ab^	10.2 ± 0.29^Aa^	809.3 ± 92.8^Aab^	6.83 ± 0.30^Aa^
NP_M_15	3347.7 ± 243.0^Ab^	9.44 ± 0.53^Bb^	708.9 ± 77.6^Ba^	6.00 ± 0.52^Ba^
AP_M_15	3737.7 ± 383.9^Ab^	9.98 ± 0.19^Aa^	830.1 ± 76.9^Aa^	6.84 ± 0.16^Aa^
NP_J_5	4228.1 ± 590.5^Aa^	10.2 ± 0.45^Aa^	689.0 ± 91.4^Aa^	5.49 ± 0.28^Ba^
AP_J_5	4154.1 ± 246.2^Aa^	10.2 ± 0.29^Aa^	714.5 ± 134.3^Aa^	6.33 ± 0.54^Aa^
NP_J_10	3718.2 ± 1030.1^Aa^	8.99 ± 1.45^Aa^	568.7 ± 202.9^Ba^	5.54 ± 0.65^Ba^
AP_J_10	3961.8 ± 446.1^Aa^	10.1 ± 0.23^Aa^	739.2 ± 67.8^Aa^	6.51 ± 0.42^Aa^
NP_J_15	3854.3 ± 1382.5^Aa^	8.84 ± 2.05^Aa^	647.7 ± 121.1^Ba^	5.67 ± 0.10^Ba^
AP_J_15	3544.6 ± 450.5^Aa^	9.84 ± 0.25^Aa^	818.9 ± 101.8^Aa^	6.92 ± 0.33^Aa^
NP_S_5	4190.0 ± 523.0^Aa^	10.20 ± 0.28^Aa^	751.1 ± 92.5^Ba^	5.61 ± 0.40^Ba^
AP_S_5	3962.4 ± 476.1^Aa^	10.07 ± 0.45^Aa^	874.1 ± 70.1^Aa^	6.74 ± 0.22^Aa^
NP_S_10	3952.1 ± 902.4^Aa^	9.19 ± 1.13^Aa^	552.7 ± 163.0^Bb^	5.28 ± 0.43^Ba^
AP_S_10	3179.2 ± 279.2^Bb^	9.72 ± 0.25^Aa^	719.6 ± 50.9^Ab^	6.84 ± 0.47^Aa^
NP_S_15	2439.9 ± 660.3^Ab^	7.18 ± 1.27^Bb^	494.3 ± 88.7^Bb^	4.95 ± 0.94^Ba^
AP_S_15	2918.2 ± 348.4^Ab^	9.53 ± 0.27^Aa^	691.5 ± 102.7^Ab^	6.57 ± 0.59^Aa^

Data are presented as the mean ± SD (*n* = 6). Different uppercase letters indicate significant differences between APs and NP and different lowercase letters indicate significant differences between different sediment depths (*p* < 0.05).

**Table 3:** Diversity of the 16S rRNA genes of *Methylomirabilis oxyfera-like* bacteria and *M. nitroreducens*-like archaea in the sediment samples of APs and NP in May, July, and September.

Sampling sites	*M. oxyfera-like* bacteria		*M. nitroreducens*-like archaea	
	Chao1	Shannon	Chao1	Shannon
NP_M_5	11.1 ± 4.17^Ba^	2.69 ± 0.35^Aa^	15.3 ± 5.64^Aa^	2.92 ± 0.42^Aa^
AP_M_5	15.9 ± 3.33^Aa^	2.91 ± 0.28^Aa^	9.22 ± 4.98^Aa^	2.10 ± 1.08^Ba^
NP_M_10	9.58 ± 4.06^Aa^	2.61 ± 0.47^Aa^	18.9 ± 6.55^Aa^	2.92 ± 0.16^Aa^
AP_M_10	11.1 ± 4.47^Aa^	2.41 ± 0.20^Ab^	12.1 ± 6.98^Aa^	2.36 ± 0.51^Aa^
NP_M_15	8.39 ± 2.71^Aa^	2.59 ± 0.30^Aa^	23.5 ± 5.26^Aa^	3.05 ± 0.22^Aa^
AP_M_15	11.1 ± 1.80^Aa^	2.35 ± 0.25^Ab^	13.9 ± 8.47^Ba^	2.50 ± 0.64^Aa^
NP_J_5	9.92 ± 2.58^Ba^	2.69 ± 0.24^Aa^	16.6 ± 2.16^Aa^	2.92 ± 0.14^Aa^
AP_J_5	14.7 ± 2.63^Aa^	2.84 ± 0.15^Aa^	5.20 ± 3.51^Bb^	1.58 ± 0.56^Ba^
NP_J_10	8.03 ± 2.94^Ba^	2.33 ± 0.64^Aa^	16.6 ± 2.03^Aa^	2.81 ± 0.32^Aa^
AP_J_10	14.2 ± 6.46^Aa^	2.67 ± 0.23^Aa^	11.3 ± 5.08^Ba^	2.26 ± 0.47^Aa^
NP_J_15	8.80 ± 4.29^Aa^	2.27 ± 0.77^Aa^	15.4 ± 2.67^Aa^	2.86 ± 0.18^Aa^
AP_J_15	10.2 ± 2.63^Aa^	2.32 ± 0.37^Aa^	12.6 ± 6.15^Aa^	2.36 ± 1.16^Aa^
NP_S_5	10.99 ± 2.99^Aa^	2.70 ± 0.29^Aa^	14.7 ± 5.23^Aa^	2.52 ± 0.79^Aa^
AP_S_5	12.0 ± 3.37^Aa^	2.55 ± 0.30^Aa^	7.69 ± 6.45^Ba^	1.71 ± 1.05^Ba^
NP_S_10	10.2 ± 3.50^Aa^	2.37 ± 0.51^Aa^	14.5 ± 2.13^Aa^	2.84 ± 0.08^Aa^
AP_S_10	7.74 ± 2.85^Ab^	2.26 ± 0.37^Aa^	12.8 ± 5.78^Aa^	2.62 ± 0.50^Aa^
NP_S_15	5.76 ± 1.72^Bb^	1.50 ± 0.50^Bb^	14.4 ± 4.70^Aa^	2.53 ± 0.53^Aa^
AP_S_15	9.57 ± 2.87^Aa^	2.09 ± 0.18^Aa^	11.2 ± 4.94^Aa^	2.36 ± 0.31^Aa^

Data are presented as the mean ± SD (*n* = 6). Different uppercase letters indicate significant differences between APs and NP and different lowercase letters indicate significant differences between different sediment depths (*p* < 0.05).

The NMDS analysis based on Bray–Curtis dissimilarity matrices demonstrated that both the bacterial and archaeal communities differed significantly between the ponds and among sediment depth across farming seasons. The ANOSIM analysis further confirmed that the archaeal and bacterial community compositions were statistically influenced by the pond type and sediment depth (Supplement Data S2–S5).

**Figure 3:**
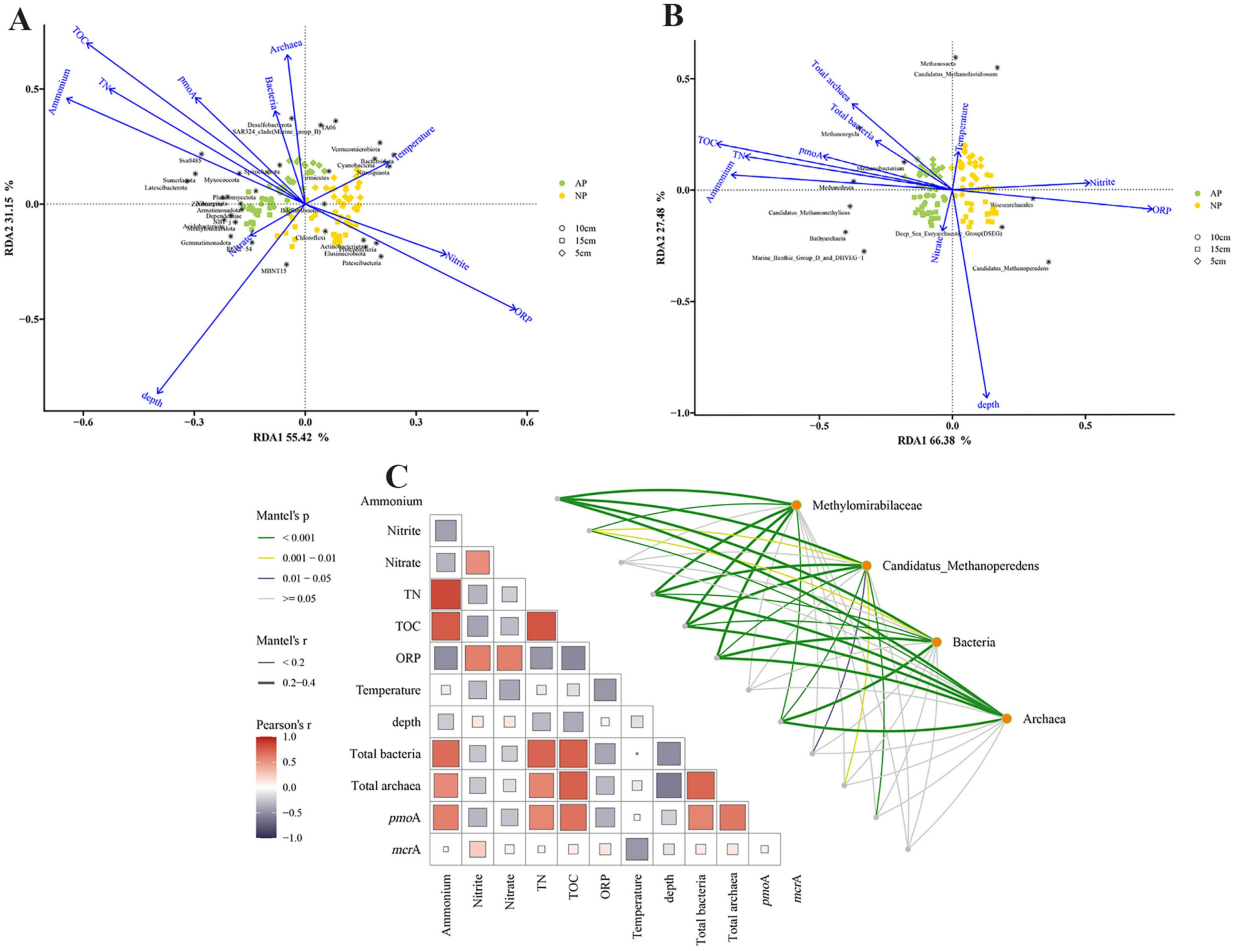
Associations between environmental factors and the composition of microbial communities. RDA analysis of environmental factors and dominant bacterial communities at phylum levels **(A)** and archaeal communities at genus levels **(B)**. Mantel tests revealed the correlation between environmental factors and the composition of bacterial, archaeal and N-/Nr-DAMO microbial communities **(C)**.

According to the Mantel tests and RDA analysis, the variations in bacterial and archaeal community structures responded differently to sediment factors. The TOC, ORP, and temperature were the dominant factors affecting bacterial community structures, while ORP and TOC were the dominant factors shaping the *M. oxyfera*-like bacterial communities. The archaeal community structures were primarily shaped by ammonia, TN, TOC, ORP, and sediment depth, whereas ammonia and nitrate mostly affected the *M. nitroreducens-like* archaea community structures (Figure 3).

### 3.4 Vertical distribution of microbial community composition

**Figure 4:**
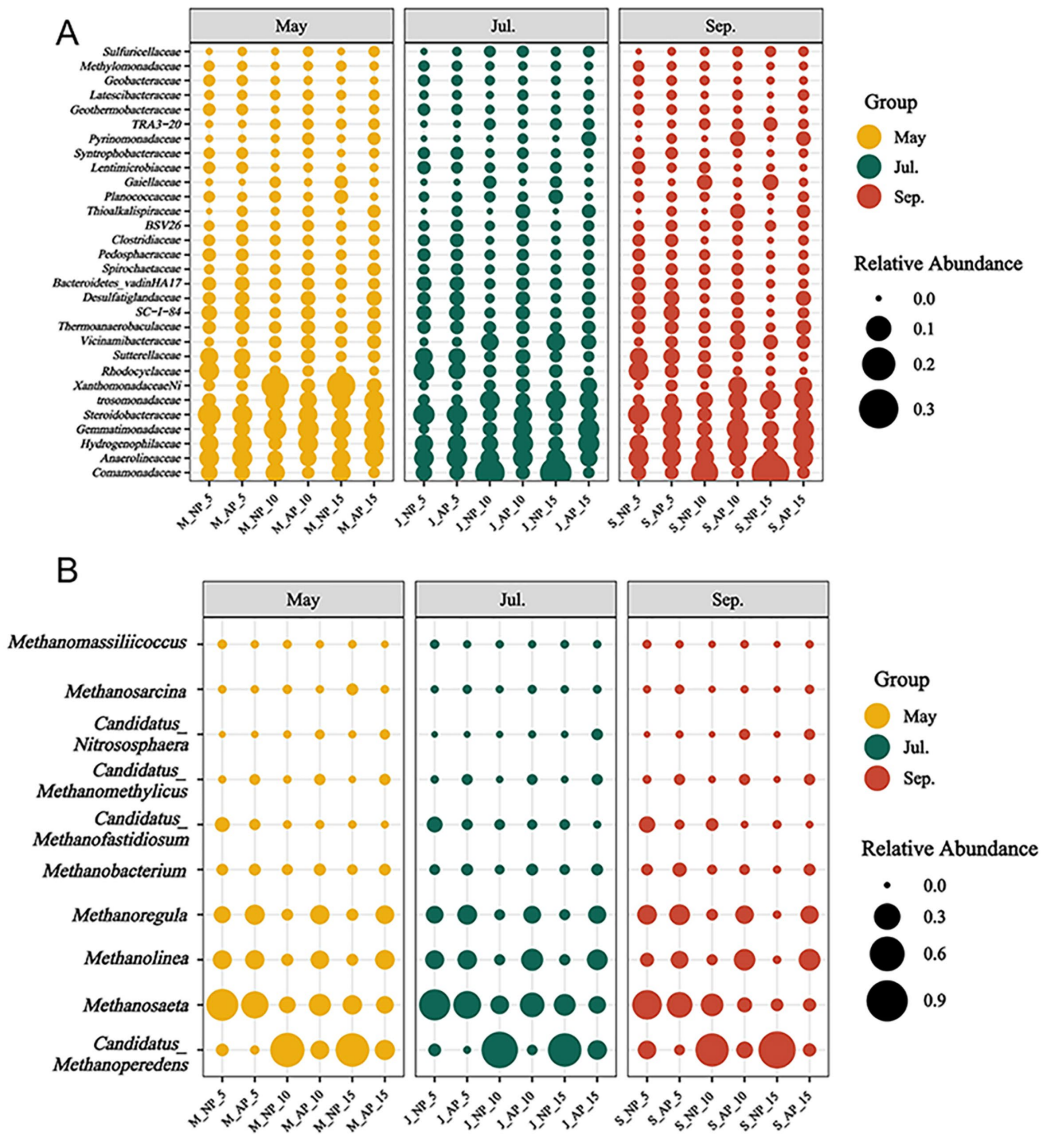
Relative abundance of top 30 bacteria at the family levels **(A)** and top 10 archaea at genus levels **(B)** communities in sediment of APs and NP in May, July and September, respectively. NP_5, NP_10 and NP_15 represent sediments in 0-5 cm, 5-10 cm and 10-15 cm layers of sediment in NP pond. AP_5, AP_10 and AP_15 represent sediments in 0-5 cm, 5-10 cm and 10-15 cm layers of sediment in APs.

The bacterial communities in APs had compositions similar to those of the NP, as well as the sedimentary layers, and were primarily composed of members of the phyla *Proteobacteria*, *Chloroflexi*, *Acidobacteria*, and *Desulfobacterota* (Figure 4). The relative abundances of *Proteobacteria* decreased, while those of *Desulfobacterota* increased in APs. The *Comamonadaceae*, *Anaerolineaceae*, and *Hydrogenophilaceae* dominated the bacterial communities. In APs, the relative abundances of *Comamonadaceae* decreased, while the *Hydrogenophilaceae* increased significantly. According to the LEfSe analysis, many taxa were enriched in APs, such as genus *Syntrophobacter*, *Haliangium*, *Desulfatiglans*, and *Sulfuricella* (May), the genus *Subgroup_18*, *Latescibacterota*, *Nitrospira*, and *Sva048*, (July), and the genus *Subgroup*_18, *Latescibacterota*, *NB1_j*, and *Sva0485* (September). The number of ASVs annotated to *Methylomirabilaceae* (*M. oxyfera*-like bacteria) displayed higher levels in 0–5 cm sediment layers (Table 1), and higher diversities of *Methylomirabilaceae* bacteria were observed in 0–5 cm sediment layers (May and July), 5–10 cm sediment layers (July), and 10–15 cm sediment layers (September) in APs. Regarding the vertical distribution, the diversities of *Methylomirabilaceae* bacteria decreased with sediment layers in May (APs) and September (both ponds) (Table 3).

Archaea sequences were primarily composed of the phyla *Halobacterota*, *Crenarchaeota*, and *Thermoplasmatota* (Figure 4). At the genus level, the distributions of most abundant genera *Candidatus_Methanoperedens*, *Methanosaeta*, and *Methanolinea* showed heterogeneity in the depth profile of the sediment. Sequences related to the genus of *Candidatus Methanoperedens* exhibited a noticeable positive trend along sediment depth, but their relative abundances were significantly decreased in APs (Table 2). In contrast, the relative abundance of *Methanosaeta* exhibited a noticeable decreasing trend as the sediment depth increased. The methanogens such as *Methanoregula*, *Methanolinea*, and *Methanobacterium* were significantly enriched in APs throughout the farming season. The diversities of *M. nitroreducens*-like archaea were significantly lower in 0–5 cm sediment layer of APs during the farming seasons (Table 3). Regarding the vertical distribution, no significant differences in the diversities of *M. nitroreducens*-like archaea were observed, except for a significantly lower indexes in the 0–5 cm sediment layers of APs in July.

### 3.5 Co-occurrence networks analysis and different environmental factors

**Figure 5:**
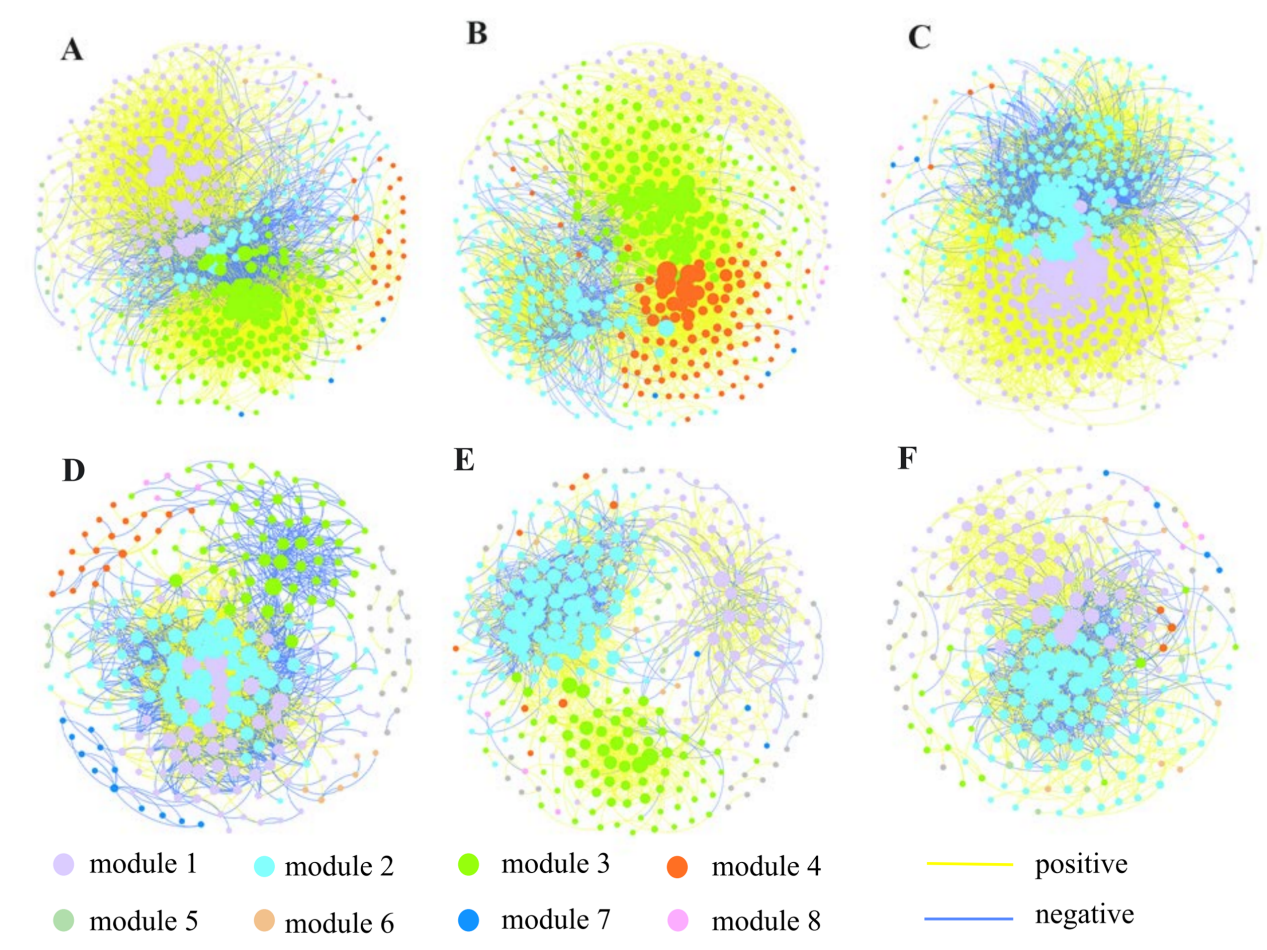
Co-occurrence network of bacterial communities in sediment of NP in May **(A)**, July **(B)** and September **(C)** and APs in May **(D)**, July **(E)** and September **(F)**. Nodes are colored by modules. The size of nodes is represented by node degree. The lines between each pair of nodes represent positive (in yellow) and negative (in blue) interactions.

**Figure 6:**
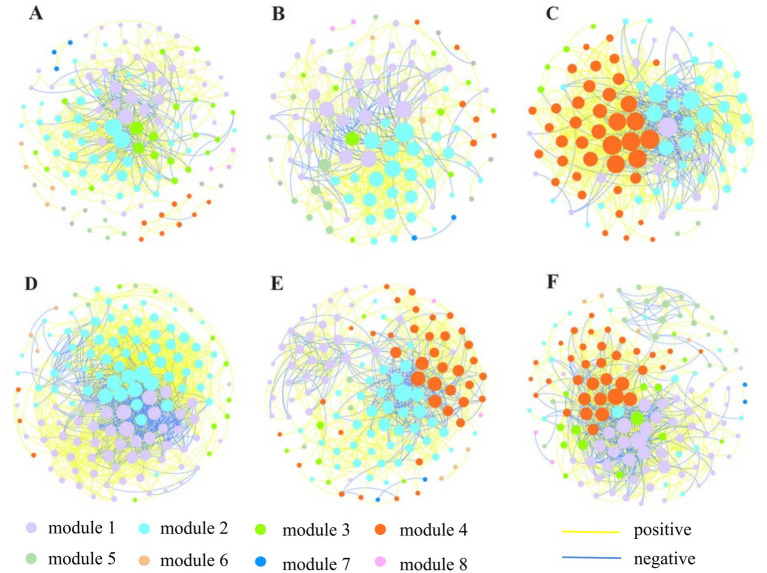
Co-occurrence networks of archaeal communities in sediment of NP in May **(A)**, July **(B)** and September **(C)** and APs in May **(D)**, July **(E)** and September **(F**). Nodes are colored by modules. The size of nodes is represented by node degree. The lines between each pair of nodes represent positive (in yellow) and negative (in blue) interactions.

To further investigate the potential inter-taxa relationships and the interaction between N/Nr-DAMO microorganisms and bacteria or archaea in the APs and NP, six bacterial (Figure 5) and six archaeal (Figure 6) co-occurrence networks were constructed using ASV data from different farming seasons. The bacterial microbial co-occurrence networks showed marked differences between the APs and NP (Table 4). The NP sediment had larger numbers of nodes and edges, higher average degree, greater modularity, and more positive edges, indicating a more connected and stable bacterial network structures. There were four (NP) and 0–4 (APs) ASVs of *M. oxyfera-like* bacteria that participated in the co-occurrence networks, and the average degrees were 16.5–42.5 (NP) and 0–12.0 (APs), respectively (Table 4). As for the archaea, the APs had larger numbers of nodes, edges, and degrees in the networks, indicating more connected and stable network structures. However, the percentage of positive correlations and the number of *M. nitroreducens-like* archaea participants in the co-occurrence networks associated with APs (4–7) were lower than those associated with the NP (23–38), indicating more competition in the archaeal co-occurrence networks and the disadvantage of *M. nitroreducens-like* archaea in APs (Table 4).

**Table 4:** Topological properties of the empirical species-species co-occurrence networks of bacterial and archaeal communities and their associated random network.

	Bacteria						Archaea					
Network properties	M_NP	M_AP	J_NP	J_AP	S_NP	S_AP	M_NP	M_AP	J_NP	J_AP	S_NP	S_AP
Nodes	513	277	512	342	483	248	127	141	105	132	99	144
Average degree	24.0	14.3	18.6	11.1	42.5	11.9	8.71	19.2	9.73	11.6	15.5	12.1
Edges	6,161	1,980	4,761	1,897	10,270	1,472	553	1,354	511	768	766	869
Positive edges	74.9%	48.2%	88.6%	57.9%	71.7%	53.3%	70.9%	68.7%	75.7%	63.5%	72.8%	56.5%
Negative edges	25.1%	51.8%	11.4%	42.1%	28.3%	46.7%	29.1%	31.3%	24.3%	36.5%	27.2%	43.5%
Module	10	15	8	19	9	16	11	6	11	8	5	8
Modularity	0.39	0.30	0.44	0.53	0.22	0.31	0.32	0.23	0.30	0.41	0.26	0.32
No. of DAMO microbes	4	0	4	4	4	2	38	7	23	4	25	7
Average degree of DAMO microbes	35.0	0	16.5	12.0	42.5	1.0	14.5	6.29	7.43	1.0	9.4	3.0

Keystone taxa were identified from bacterial communities based on the criteria of (Yu et al., 2023). The bacterial keystone taxa were more diverse in the NP (47 ASVs) than the APs (11 ASVs). In contrast, keystone taxa of the archaeal community demonstrated more diversity in the APs. The keystone ASVs belonging to the genus *Candidatus Methanoperedens* were found only in NP. In APs, the archaeal keystone taxa were included by members of *Bathyarchaeia*, *Marine_Benthic_Group_D_and_DHVEG-1*, *Woesearchaeales*, *Candidatus Nitrososphaera*, and methanogens such as genus *Methanosaeta*, *Methanoregula*, and *Methanolinea*.

## 4 Discussion

The composition of bacterial–archaeal communities is a key feature of aquaculture sediment because microbes provide multiple essential ecosystem services. In APs, artificial management, especially the increases in stocking density and feed input, has been applied to enhance production and economic benefits (Bakar et al., 2015; Li R. et al., 2025; Li X. et al., 2025). The inorganic nutrients originating from residual feeds and excretion stimulate organic carbon production through phytoplankton photosynthesis. The uneaten feed, dead plankton, and the excrement of culture species settle on the pond sediment, leading to higher amounts of organic matter load (Li R. et al., 2025; Liao et al., 2025). This organic matter provides a potential energy source for microbial communities, including those involved in CH_4_ cycling, allowing them to proliferate and spread quickly. Previous studies have shown that the symbiotic network between methanogens and methanotrophs becomes increasingly competitive and complex, and methanogens occupy central roles and appear to hold a superior position relative to methanotrophs, resulting in a source of CH_4_ in sediments of AP (Liu et al., 2024). The human-induced contrasting sediment conditions for microbial communities may also determine the niche differentiation of N-/Nr-DAMO microbes in APs.

### 4.1 Spatiotemporal distributions of sediment prokaryotic abundances and communities

The abundances of total bacteria and archaea were found to increase significantly in AP sediments compared with the natural succession pond sediments (no human intervention), demonstrating relatively high copy numbers compared with previous studies in lakes (Sherysheva and Reznikova, 2023), paddy fields (Tang et al., 2021), wetlands (Ji et al., 2021), and reservoirs (Kosolapov, 2020). In addition, higher archaeal diversities were observed in APs. According to the correlation analysis, TOC, TN, and NH_4_^+^ were the main factors affecting bacterial and archaeal abundances and α-diversities. Higher microbial copy numbers and diversities are usually positively correlated with the microbial capacity to respond to resource availability (Liu et al., 2024; Nair et al., 2018; Viso et al., 2024). The rich sediment content originating from endogenous (e.g., plankton) and exogenous (e.g., feed) sources favors the growth of bacteria and archaea, especially in surface sediment (Dai et al., 2018; Li et al., 2017; Wu et al., 2026). The vertical distribution of total bacteria and archaea demonstrated similar abundance and diversity patterns across the NP and APs. The bacterial α-diversities were significantly higher in 0–5 cm sediment layers. The archaeal α-diversities increased along with vertical sediment layers in May, while they were significantly higher in 0–5 cm sediment layers in September. In APs, surface sediment accumulated available organic matter from the uneaten feed, dead plankton, and excrement of culture species throughout the farming season, thereby supporting highly diverse microbial communities (Dai et al., 2018; Hou et al., 2022; Wu et al., 2026). Additionally, the archaeal gene abundances were lower than those of bacteria in almost all samples. Bacteria exhibit higher environmental tolerances and a broader range of physiological characteristics than archaea, making them successful colonists in sediment environments (Wang et al., 2021; Yu et al., 2023).

In line with earlier studies, the vertical profiles and the aquaculture practices significantly influenced the relative abundances of certain bacterial groups (Li R. et al., 2025; Li X. et al., 2025; Lin and Lin, 2022; Resende et al., 2015). Two big clusters of correlations were observed between the phylum abundance and sediment properties. One cluster was formed by phyla shaped by ORP, such as *Proteobacteria*, *Chloroflexi*, and *Bacteroidota.* For example, in the main lineages of phylum *Proteobacteria*, the dominant bacterial families *Comamonadaceae* and *Nitrosomonadaceae* decreased, whereas the *Hydrogenophilaceae* increased significantly in APs. Most species of *Comamonadaceae* and *Nitrosomonadaceae* have aerobic heterotrophic metabolisms and play crucial roles in organic matter mineralization and nitrification in aquatic sediments (Dyksma et al., 2016; Figueroa et al., 2021). The *Hydrogenophilaceae* are dominant flora in anaerobic ammonia oxidation and autotrophic desulfurization–denitrification (Chen et al., 2016). The anaerobic conditions may provide a suitable environment for the accumulation of *Hydrogenophilaceae* in APs. The second cluster, composed of *Acidobacteriota*, *Desulfobacterota*, and *Gemmatimonadota*, increased with nutrient availability (TOC, TN, and NH_4_^+^) and sediment depth. These phyla were reported to play important roles in metabolizing complex organic matter and producing small organic acids, including methanogenic precursors such as acetate, and the denitrification and desulfurization contributing to ecosystem stability (Li R. et al., 2025; Li X. et al., 2025; Richy et al., 2024). The microbial enrichment indicates the presence of active carbon metabolic processes in aquaculture sediments. These results suggested that nutrient accumulation and decreases in redox potential lead to a corresponding reduction in the quantity of nitrifying bacteria, accompanied by an increase in functional taxa involved in organic carbon metabolism and denitrification in APs (Figueroa et al., 2021; Richy et al., 2024).

### 4.2 Spatiotemporal distributions of sediment N-/Nr-DAMO microbial abundances and communities

In the present study, the abundances of N-DAMO bacterial *pmoA* gene in aquaculture sediment were higher than those in the natural succession ponds and Taihu Lake (1.82 × 10^4^–6.11 × 10^6^, Ding et al., 2022). The values were approximately equal to those found in the paddy fields (2.5 × 10^6^–1.07 × 10^8^ copies/g, Wang et al., 2021), river sediments (1.3 × 10^6^–1.5 × 10^7^ copies/g, Cheng et al., 2022), and freshwater wetlands (1.6 × 10^6^–3.2 × 10^7^ copies/g, Shen et al., 2017; Hu et al., 2014). In addition, the *M. oxyfera*-like bacteria in APs exhibited significantly higher diversities compared with the natural succession ponds. The higher organic carbon content and anoxic environment (lower ORP) provide favorable conditions for methanogenesis in APs, which may provide a sufficient carbon source for the growth of *M. oxyfera*-like bacteria (Zhang et al., 2024; Wang et al., 2026). Furthermore, the *M. oxyfera*-like bacterial community structure and N-DAMO bacterial *pmoA* were affected by NH_4_^+^ concentration in sediment. NH_4_^+^ is oxidized to NO_2_^−^ through nitrification, which increases the availability of electron acceptor for *M. oxyfera*-like bacteria (Wang et al., 2026; Yang et al., 2024). The *M. oxyfera*-like bacterial *pmoA* gene abundances exhibited no spatial or temporal heterogeneity along the aquaculture sediment profile (0–15 cm), which contrasts with the previous observations (Hui et al., 2017; Ding et al., 2022). Such a disparity could be explained by the fact that resource limitation was no longer the main driver of microbial communities when nutrients were plentiful, and the living environment was moderate (Hu et al., 2025; Yu et al., 2023).

As the dominant archaeal phyla, *Halobacterota* was mostly composed of the genera *Candidatus Methanoperedens*, *Methanosaeta*, *Methanolinea*, and *Methanoregula* (Bongoua-Devisme et al., 2024; Bräuer et al., 2020), which exhibited fine-scale vertical heterogeneity in aquaculture sediments. The genus *Candidatus Methanoperedens* (*M. nitroreducens*-like archaea) employs MCR to activate CH_4_ that subsequently oxidizes to CO_2_ through “a reverse methanogenesis” pathway (Zehnle and Schoelmerich, 2025; Haroon et al., 2013), increasing the lineage in the deeper-layer sediment, but decreasing in APs. The results align with previous studies showing that hydrogenotrophic methanogens (*Methanolinea* and *Methanoregula*) dominated the archaeal communities in aquaculture sediments, as the H_2_ and CO_2_ production increased by the oxidation of fatty acids from residual gathering (Liu et al., 2024). The hypoxia in APs resulted in methanogenesis becoming the dominant terminal process of TOC degradation, which has been proven to stimulate methanogenic activity over CH_4_ oxidation (Wang et al., 2026). Moreover, the potential electron acceptors—e.g., nitrate—increased along sediment depth in natural succession ponds, which created favorable conditions for *M. nitroreducens*-like archaea (Shen et al., 2018; Yang et al., 2024). In aquaculture ponds, the organic carbon loading promoted heterotrophic denitrifiers to consume NO_3_^−^ faster (Jiang et al., 2025), which may put *M. nitroreducens*-like archaea at a disadvantage in the competition for electron acceptors, and the reduction in nitrate concentration may act as a limiting factor for the growth of *M. nitroreducens*-like archaea (Hu et al., 2025; Fan et al., 2020).

### 4.3 Co-occurrence patterns of the prokaryotic communities in sediments during farming seasons

The co-occurrence networks were constructed separately to elucidate the distinctions of microbial interactions in APs and the natural succession ponds. The bacteria seem more interconnected because they have more nodes and edges than the archaeal network; this may be attributed to higher bacterial diversity and more ecological niches for bacteria (Chen and Wen, 2021; Yu et al., 2023). The results showed more bacterial network complexities with a larger number of nodes, edges, and average degrees, but fewer modules in the natural succession pond than in the APs. The aquaculture practices promote the bacterial habitat heterogeneity, different selection mechanisms, and a cluster of closely related species (Beman et al., 2011; Viso et al., 2024; Ye et al., 2009). In the NP, 47 ASVs were identified as the bacterial keystone taxa, mostly belonging to *Nitrosomonadaceae*, which are associated positively and closely (higher degree) with other bacterial taxa. In the APs, *Hydrogenophilaceae* were identified as the most abundant keystone species among 11 ASVs, and *Nitrosomonadaceae* turned out to be in competition with other species. The *M. oxyfera*-like bacteria were synergistic with taxa (e.g., *Bacteroidetes_vadinHA17*, *SC-I-84*, and *Nitrosomonadaceae*) in natural succession pond, whereas these taxa appeared to be in disadvantageous positions in the co-occurrence networks of APs, which may lead to fewer *M. oxyfera*-like bacteria participating in the bacterial co-occurrence networks.

Regarding archaea, a higher degree of network complexity and modularity was detected in APs, suggesting that the populations within the archaeal communities may possess a more comparable modular structure. The methanogens dominated the archaeal community structure, and the higher abundance, diversity, and connectivity of the methanogens may explain the CH_4_ hot spot in the aquaculture sediment (Fan et al., 2019; Liu et al., 2024). In NP, the ASVs belonging to *M. nitroreducens*-like archaea were identified as the most abundant keystone taxon in May and September, which demonstrated close interaction with other archaeal communities, especially members of *Bathyarchaeia*. This observation may indicate the reliance of these microbes on collaboration, which can boost their ability to adapt to environmental selection (Yu et al., 2023). Whereas, the members of *Bathyarchaeia* occupied a large proportion of keystone species in APs, which demonstrated the negative correlations with *M. nitroreducens*-like archaea. Recent studies have underscored the fermentative capabilities of dominant lineages, including members of *Bathyarchaeia*, suggesting their roles in degrading organic carbon substrates to produce acetate and alcohol (Yi et al., 2024; Wang et al., 2022). The *Bathyarchaeia* were found to be positively correlated with methanogens such as *Methanosaeta* and *Methanolinea.* These results implied that CH_4_ production may be the most indispensable link in the organic carbon cycle in APs.

Most of the interactions in the bacterial and archaeal networks were positive in natural succession ponds, suggesting that cooperative or mutualistic interactions such as symbiosis were the main types of interaction among microorganisms (Beman et al., 2011; Gong et al., 2022). In contrast, more negative links occurred in Aps, indicating antagonistic or competition correlation among species. The mutualistic and more complicated network structure stabilized communities by incorporating mixed interaction types and enhancing resistance to environmental disturbance (Chen and Wen, 2021; Yu et al., 2023). Even though the interactions inferred from co-occurrence networks may not occur spatially, the results of this study suggest that the communities may be less stable and more sensitive to environmental changes in APs.

## 5 Conclusion

In conclusion, the present study provided a comprehensive analysis of the spatiotemporal variation in bacterial–archaeal abundance, community composition, diversity, and aquaculture pond sediment interaction, compared with a natural succession pond. In addition, the niche differentiation of N-/Nr-DAMO microbes represented by *Methylomirabilaceae* and *Candidatus Methanoperedens* was analyzed. Generally, the abundances and microbial diversities of the total bacteria and total archaea were significantly higher in APs. The vertical profiles and the aquaculture practices significantly influenced the bacterial and archaeal community structure, which was shaped primarily by ORP and nutrient availability. In APs, bacterial co-occurrence networks were less complex, and fewer N-DAMO bacteria participating in the bacterial co-occurrence networks. The archaea (e.g. desulphurizor, denitrifiers, methanogens) showed negatively correlated with respect to the distribution of Nr-DAMO archaea which were fewer presented in the archaeal co-occurrence networks. These results appeared that the niches of N-/Nr-DAMO microbes were suppressed in aquaculture ponds.

## Data Availability

The data presented in this study are publicly available. The data can be found here: https://ngdc.cncb.ac.cn/gsa/, accession number CRA031053 for archaea and CRA031059 for bacteria.
