# Supplementary material for: Spatial and temporal variation in bacterial–archaeal community and niche differentiation of denitrifying anaerobic methane-oxidizing microbes in grass carp (Ctenopharyngodon idellus) aquaculture ponds of Northern China
Source: Front Microbiol. 2026 Apr 10;17:1782664. doi: 10.3389/fmicb.2026.1782664 (PMC13106468; doi:10.3389/fmicb.2026.1782664)
Supplement: Supplementary file 2 [file Supplementary_file_1.docx]

Table S1 The qPCR reaction system of total bacteria and total archaea, N-DAMO bacterial *pmoA* and Nr-DAMO archaeal *mcrA* in sediments.

|  | Primer | Sequence (5'-3') | PCR reaction condition |
| --- | --- | --- | --- |
| *Total bacteria* | 515F | GTGCCAGCMGCCGCGG | Predenaturation at 95 *°C* for 30*s*, 40× (denaturation at 95 *°C* for 5*s*, annealing at 56 *°C* for 30*s*, cycle number of 40, extension at 75 *°C* for 15*s*), extension at 72*°C* for 2 *min* |
|  | 907R | CCGTCAATTCMTTTRAGTTT |  |
| *Total archaea* | Ar109F | ACKGCTCAGTAACACGT | Predenatured at 95 *°C* for 30*s*, 40× (denatured at 95 *°C* for 10*s*, annealed at 55 *°C* for 30*s*, extended at 75 *°C* for 15*s*, cycle number 40), extended at 72 *°C* for 2 *min* |
|  | Ar344R | TCGCGCCTGCTGCTCCCCGT |  |
| N-DAMO bacterial *pmoA* | cmo182 | TCACGTTGACGCCGATCC | Predenaturation at 95 *°C* for 2 *min*, 40 × (denaturation at 95 *°C* for 10*s*, annealing at 60 *°C* for 50*s*, extension at 75 *°C* for 40*s*), extension at 72 *°C* for 2 *min* |
|  | Cmo568 | GCACATACTCCATCCCCATC |  |
| Nr-DAMO archaeal *mcrA* | McrA159F | AAAGTGCGGAGCAGCAATCACC | Predenaturation at 95 *°C* for 2 *min*, 40 × (denaturation at 95 *°C* for 10*s*, annealing at 57 *°C* for 30*s*, extension at 75 *°C* for 40*s*), extension at 72 *°C* for 2 *min* |
|  | McrA345R | TCGTCCCATTCCTGCTGCATTGC |  |

In the 20 *μL* qPCR reaction system, 10 *μL* TB Green premix (TaKaRa, Japan), 0.4 *μL* ROX Reference Dye (50x), 0.4 *μL* pre-primer (10 *μM*), 0.4 *μL* post primer (10 *μM*), 2 *μL* DNA template, 6.8 *μL* enzyme-free water.

Table S2 Two-way ANOVA of TN, NO_3_^-^, NO_2_^-^, NH_4_^+^, TOC and ORP of APs and NP in different sediment depth in May, July and September.

| Dependent variable | Factors | May | | | Jul. | | | Sep. | | |
| --- | --- | --- | --- | --- | --- | --- | --- | --- | --- | --- |
|  |  | df | F | p | df | F | p | df | F | p |
| TN | depth | 2 | 2.32 | 0.12 | 2 | 26.0 | 0.00^*^ | 2 | 16.2 | 0.00^*^ |
|  | pond | 1 | 27.1 | 0.00^*^ | 1 | 302.0 | 0.00^*^ | 1 | 107.6 | 0.00^*^ |
|  | depth × pond | 2 | 1.16 | 0.33 | 2 | 5.49 | 0.00^*^ | 2 | 1.18 | 0.32 |
| NO_3_^-^ | depth | 2 | 0.67 | 0.52 | 2 | 5.02 | 0.01^*^ | 2 | 11.9 | 0.00^*^ |
|  | pond | 1 | 8.27 | 0.01^*^ | 1 | 5.49 | 0.02^*^ | 1 | 132.0 | 0.00^*^ |
|  | depth × pond | 2 | 0.10 | 0.90^*^ | 2 | 2.56 | 0.09 | 2 | 11.2 | 0.00^*^ |
| NO_2_^-^ | depth | 2 | 0.74 | 0.49 | 2 | 0.85 | 0.44 | 2 | 5.94 | 0.01^*^ |
|  | pond | 1 | 2.06 | 0.16 | 1 | 6.69 | 0.02^*^ | 1 | 68.4 | 0.00^*^ |
|  | depth × pond | 2 | 0.07 | 0.93 | 2 | 0.03 | 0.97 | 2 | 7.45 | 0.00 |
| NH_4_^+^ | depth | 2 | 4.14 | 0.03^*^ | 2 | 0.30 | 0.74 | 2 | 18.5 | 0.00^*^ |
|  | pond | 1 | 25.3 | 0.00^*^ | 1 | 194.1 | 0.00^*^ | 1 | 126.3 | 0.00^*^ |
|  | depth × pond | 2 | 7.86 | 0.02^*^ | 2 | 0.34 | 0.71 | 2 | 1.45 | 0.25 |
| TOC | depth | 2 | 32.1 | 0.00^*^ | 2 | 118.7 | 0.00^*^ | 2 | 35.0 | 0.00^*^ |
|  | pond | 1 | 246.0 | 0.00^*^ | 1 | 952.1 | 0.00^*^ | 1 | 320.1 | 0.00^*^ |
|  | depth × pond | 2 | 1.05 | 0.00^*^ | 2 | 2.18 | 0.00^*^ | 2 | 6.67 | 0.00^*^ |
| ORP | depth | 2 | 3.52 | 0.04^*^ | 2 | 34.9 | 0.00^*^ | 2 | 32.0 | 0.00^*^ |
|  | pond | 1 | 2.98 | 0.1 | 1 | 319.5 | 0.00^*^ | 1 | 1678.5 | 0.00^*^ |
|  | depth × pond | 2 | 0.15 | 0.87 | 2 | 55.9 | 0.00^*^ | 2 | 102.2 | 0.00^*^ |

Table S3 Two-way ANOVA of total bacteria, total archeae, N-DAMO bacterial *pmoA*, and Nr-DAMO archaeal *mcrA* abundance of AP and NP in different sediment depth in May, July and September.

| Dependent variable | Factors | May | | | Jul. | | | Sep. | | |
| --- | --- | --- | --- | --- | --- | --- | --- | --- | --- | --- |
|  |  | df | F | p | df | F | p | df | F | p |
| Total bacteria | depth | 2 | 12.8 | 0.00^*^ | 2 | 27.2 | 0.00^*^ | 2 | 32.1 | 0.00^*^ |
|  | pond | 1 | 40.0 | 0.00^*^ | 1 | 112.5 | 0.00^*^ | 1 | 41.3 | 0.00^*^ |
|  | depth × pond | 2 | 1.73 | 0.20 | 2 | 4.62 | 0.02^*^ | 2 | 1.21 | 0.31 |
| Total archaea | depth | 2 | 18.3 | 0.00^*^ | 2 | 49.7 | 0.00^*^ | 2 | 17.7 | 0.00^*^ |
|  | pond | 1 | 40.5 | 0.00^*^ | 1 | 80.4 | 0.00^*^ | 1 | 16.3 | 0.00^*^ |
|  | depth × pond | 2 | 2.06 | 0.15 | 2 | 9.98 | 0.00^*^ | 2 | 2.66 | 0.086 |
| N-DAMO bacterial *pmoA* | depth | 2 | 2.07 | 0.14 | 2 | 3.37 | 0.05^*^ | 2 | 1.07 | 0.36 |
|  | pond | 1 | 15.2 | 0.00^*^ | 1 | 60.8 | 0.00^*^ | 1 | 6.98 | 0.013^*^ |
|  | depth × pond | 2 | 0.37 | 0.70 | 2 | 2.11 | 0.14 | 2 | 0.50 | 0.61 |
| Nr-DAMO archaeal *mcrA* | depth | 2 | 1.01 | 0.38 | 2 | 0.98 | 0.39 | 2 | 3.04 | 0.06 |
|  | pond | 1 | 0.45 | 0.51 | 1 | 6.74 | 0.01^*^ | 1 | 7.69 | 0.01^*^ |
|  | depth × pond | 2 | 2.34 | 0.11 | 2 | 3.70 | 0.04^*^ | 2 | 1.45 | 0.25 |

Table S4 Two-way ANOVA of α diversity indexes of bacteria, archeae, *M. oxyfera-like* bacteria and *M. nitroreducens*-like archaea in APs and NP in different sediment depth in May, July and September.

| Dependent variable | Factors | May | | | Jul. | | | Sep. | | |
| --- | --- | --- | --- | --- | --- | --- | --- | --- | --- | --- |
|  |  | df | F | p | df | F | p | df | F | p |
| Bacteria | | | | | | | | | | |
| Chao1 | pond | 1 | 21.1 | 0.00^*^ | 1 | 0.03 | 0.86 | 1 | 0.84 | 0.37 |
|  | depth | 2 | 14.2 | 0.00^*^ | 2 | 1.22 | 0.31 | 2 | 18.5 | 0.00^*^ |
|  | depth × pond | 2 | 0.69 | 0.51 | 2 | 0.37 | 0.70 | 2 | 3.63 | 0.04^*^ |
| Shannon | pond | 1 | 17.5 | 0.00^*^ | 1 | 3.79 | 0.06 | 1 | 13.68 | 0.00^*^ |
|  | depth | 2 | 8.85 | 0.00^*^ | 2 | 2.19 | 0.13 | 2 | 17.52 | 0.00^*^ |
|  | depth × pond | 2 | 1.24 | 0.30 | 2 | 1.00 | 0.38 | 2 | 8.94 | 0.00^*^ |
| Archaea | | | | | | | | | | |
| Chao1 | pond | 1 | 5.98 | 0.02^*^ | 1 | 8.32 | 0.01^*^ | 1 | 23.3 | 0.00^*^ |
|  | depth | 2 | 4.74 | 0.02^*^ | 2 | 1.18 | 0.32 | 2 | 16.0 | 0.00^*^ |
|  | depth × pond | 2 | 0.85 | 0.44 | 2 | 1.31 | 0.29 | 2 | 0.41 | 0.67 |
| Shannon | pond | 1 | 46.5 | 0.00^*^ | 1 | 52.03 | 0.00^*^ | 1 | 60.7 | 0.00^*^ |
|  | depth | 2 | 9.88 | 0.00^*^ | 2 | 2.59 | 0.09 | 2 | 1.82 | 0.18 |
|  | depth × pond | 2 | 5.11 | 0.01^*^ | 2 | 0.75 | 0.48 | 2 | 0.71 | 0.50 |
| *M. oxyfera-like* bacteria | | | | | | | | | | |
| Chao1 | pond | 1 | 6.41 | 0.02^*^ | 1 | 10.3 | 0.00^*^ | 1 | 0.69 | 0.42 |
|  | depth | 2 | 3.95 | 0.03^*^ | 2 | 1.58 | 0.22 | 2 | 5.34 | 0.01^*^ |
|  | depth × pond | 2 | 0.67 | 0.52 | 2 | 1.18 | 0.32 | 2 | 3.40 | 0.04^*^ |
| Shannon | pond | 1 | 0.46 | 0.50 | 1 | 1.35 | 0.26 | 1 | 0.75 | 0.39 |
|  | depth | 2 | 3.87 | 0.03^*^ | 2 | 3.15 | 0.06 | 2 | 14.9 | 0.00^*^ |
|  | depth × pond | 2 | 1.85 | 0.17 | 2 | 0.31 | 0.74 | 2 | 3.65 | 0.04^*^ |
| *M. nitroreducens*-like archaea | | | | | | | | | | |
| Chao1 | pond | 1 | 12.3 | 0.00^*^ | 1 | 24.8 | 0.00^*^ | 1 | 0.73 | 0.49 |
|  | depth | 2 | 3.01 | 0.06 | 2 | 2.50 | 0.10 | 2 | 5.50 | 0.03^*^ |
|  | depth × pond | 2 | 0.24 | 0.79 | 2 | 3.84 | 0.03^*^ | 2 | 0.86 | 0.43 |
| Shannon | pond | 1 | 10.6 | 0.00^*^ | 1 | 17.1 | 0.00^*^ | 1 | 2.89 | 0.07 |
|  | depth | 2 | 0.61 | 0.55 | 2 | 1.28 | 0.29 | 2 | 3.64 | 0.06 |
|  | depth × pond | 2 | 0.19 | 0.83 | 2 | 1.94 | 0.16 | 2 | 0.94 | 0.40 |
